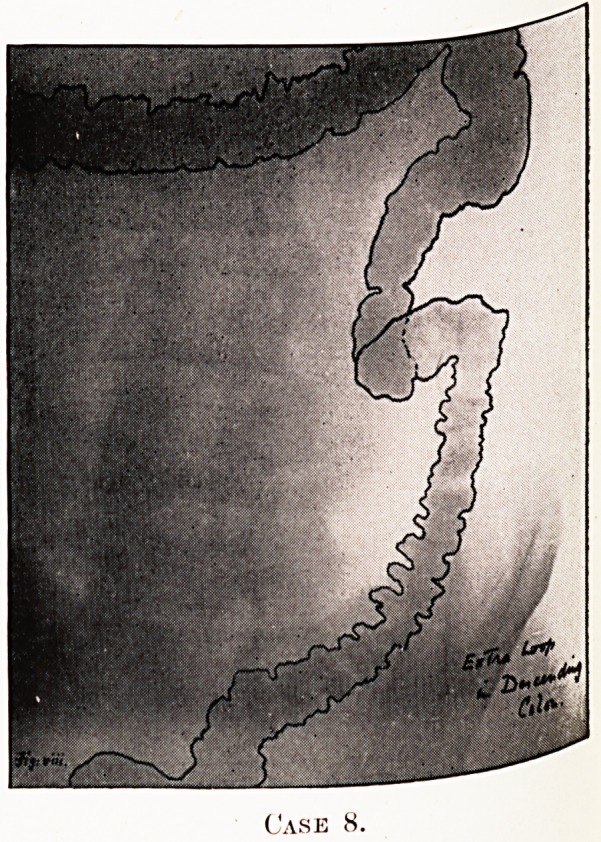# Redundant Colon: A Group of Cases Exhibiting Symptoms Traceable to This Condition

**Published:** 1928

**Authors:** Gilbert B. Bush

**Affiliations:** Assistant Radiologist, Bristol General Hospital; Recognised Teacher in Radiology, University of Bristol


### REDUNDANT COLON: A GROUP OF CASES EXHIBITING SYMPTOMS TRACEABLE TO THIS CONDITION.

BY

GILBERT B. BUSH, B.A., M.B., CH.B., D.M.R.E. (Cantab.),

*Assistant Radiologist, Bristol General Hospital ; Recognised Teacher in Radiology, University of Bristol.*

---

THE normal adult colon has an average length of 2 metres, and if longer than this cannot fit into the body without forming extra loops, reduplications or even kinks and angulations. Being mobile, these loops need not be fixed or constant in position, nor need they give rise to any symptoms *provided the colon is healthy and functioning normally and regularly.*

With regard to their etiology, Kantor,[1] who has studied nearly 700 cases, considers that they are congenital, and even hereditary and familial in their transmission. But it is probable that this is by no means the whole truth, and that in many cases the condition is acquired.

From 9 to 14 per cent. of all people show these redundancies, and the incidence among constipated people is as high as 23 per cent. (Kantor). They are not generally associated with visceroptosis, being commoner in the "sthenic," active, strong type of person.

All cases show a predisposition to imperfect colonic function, and 77 per cent. of Kantor's cases had long-standing constipation; the greater the looping the greater the constipation.

Other symptoms which can often be traced to the presence of these redundancies, when associated with impeded function, are: (1) *vague abdominal discomfort* (all cases in this series); (2) *irregular pain*, often of a colicky nature due to commonly associated spasm (Cases 2, 3, 4, 7 and 8); (3) *gas distress* (all cases); (4) *precordial pain and palpitation* following gas-collection under the left leaf of the diaphragm (Cases 3 and 4); (5) *belching or hiccough* and epigastric pain with discomfort after meals due to the same cause as (4) and to the misdirected efforts of the patient to rid himself of gas that seems to be in the stomach (Cases 4 and 6); (6) *mucous stools* (Cases 2, 4 and 6), occasionally with blood (Case 6); (7) *alternating constipation and diarrhœa* (Cases 2, 4 and 7); (8) *vomiting* (Case 7); (9) *mental depression, carcinophobia, etc.* (Cases 4, 6 and 7); (10) *rectal symptoms* from portal congestion and hæmorrhoids (Cases 5, 6 and 7).

These symptoms are not due to the mere presence of the redundancy, but to associated spasm, atony and stasis, and to the fermentation, excessive gas-formation and toxic absorption resulting from the latter.

A redundancy in the distal part of the colon may produce symptoms localised in the proximal half, suggestive of appendicitis or of biliary colic, due to a "throw-back" of the fæcal column and retention.

*The diagnosis* is suggested by (*a*) a long history of constipation (this was not present in Case 8); (*b*) a long interval between stools; (*c*) occasional enormous evacuations. It can be positively confirmed by an opaque-meal X-ray examination, followed by an opaque enema for preference.

Before discussing the treatment of the condition there are a few points that need clearing up in connection with its pathology. Are such redundant loops in any way causal or contributory to the symptoms so often found associated with them, or are they rather effects or negligible by-products of colon mal-function in a congenitally long colon? In a healthy colon a "loop" is not a fixed or static condition, but obviously if it become overfilled, displaced or distorted it can impede the forward motion of the fæcal column by (1) kinking and by (2) interference with local blood supply, producing irregular neuro-muscular action and abnormal mucosal function. The latter shows itself in a gradual loss of normal neuro-muscular propelling power, a tendency to spasm and colic, and a deficient absorption of $CO_2$ and $CH_4$ into the blood stream, thus augmenting the commonest symptom, viz. gas-distress. Note the presence of gas distal to the loops in all the cases illustrated (except Case 1 and Case 8, who had an opaque enema). Moreover, if the state of the musculature of the loop become so atrophic that the loop becomes practically a passive tube, then we have the condition of a regular plumber's trap, a local cesspool, the contents of which include a residue that is changed less and less and tends to increase.

Once colon mal-function begins, then, in a person having a congenitally long colon, the redundant loop becomes highly important as a link in the chain of cause and effect, and a vicious circle is very easily induced.

*Treatment.*—Surgical measures are contra-indicated except in the rare cases where volvulus has occurred or where definite evidence of adhesions or marked obstruction is found. (The mobility of the loop can be tested under the X-rays by systematic palpation, and its emptying rate observed.)

In the attempt to restore normal colonic function the presence of the redundant loops is a complication which was most satisfactorily met in the majority of this series of cases by judicious abdominal massage and exercises, the masseur regulating his manipulations by constant reference to a skiagram showing the exact anatomy of the colon in question. Cathartic and enema-habits should be suspended, a rough bulky diet and plenty of water should be prescribed, together with lubrication by oil taken by mouth after meals and, if it can be retained, *per rectum* at night (6 oz.), in the early stages. If spasticity is present, increasing doses of belladonna are valuable (10 to 25 minims t.d.s.).

In these cases the very fact of relieving the patient's mind by assuring him of the absence of serious disease is a powerful factor in successful treatment. The condition can be explained to him, and he should be told that in his case it is not necessary for the bowels to move every day. In Case 6 the effect of this line of treatment was dramatic : after a month's treatment he was a totally different man. Intelligent patients can be shown by the masseur how to massage their own abdomens and what exercises to perform, so that the need of prolonged special treatment is dispensed with.

Relapses may occur, and every effort should be made to prevent the patient from becoming introspective, and to encourage him to take regular exercise and to interest himself in things outside his own ego. The vicious circle can thus be broken and normal colonic function restored, but it must always be remembered that the presence of such an abnormality

### PLATE IV.

CASE 1.

CASE 2.

CASE 3.

CASE 4.

PLATE V.

Case 4.

Case 6.

Case 7.

Case 8.

in the colon constitutes a permanent disability and increases the liability to relapse.

*Notes on the Cases and Illustrations.* (*All Opaque Meals except Case* 8.)

In the appended illustrations the colon has been outlined in Indian ink solely in order to show more clearly, in the reproductions, the *main direction* taken by the loops, some of them lying in several different planes only recognisable on screen examination at different angles.

**Cases 1 and 2.**—Long dependent transverse colons with no loops, but in Case 2 causing symptoms. Case 2 had coloptosis of proximal half, " splenic drag " and gas-distress.

**Case 3.**—A complication of the condition seen in Case 1. Angulated loop in transverse colon. Proximal retention, spasm and distal gas-distress.

**Case 4.**—(Figs. 4 and 5.)—Proximal retention, marked gas-distress, colic.

**Case 6.**—Marked degree of redundancy. " Fulness " and gas-distress, no pain, but intense depression, loss of weight and lack of interest in life.

**Case 7.**—Indigestion for years (though stomach and duodenum found to be normal). Hiccough. Pain in left hypochondrium. Constipated. Occasional vomiting.

**Case 8.**—(Enema.)—Recurrent attacks of pain, sometimes severe, under left costal margin. Very active man, always healthy, bowels regular. No vomiting or nausea.

### SUMMARY.

1. The anatomy, etiology and incidence of redundant colon are described.

2. Symptoms traceable to this condition in a mal-functioning colon as observed in the cases selected are detailed.

3. The question as to how far the condition can be considered pathological is discussed.

4. The treatment is outlined.

5. Notes and illustrations of seven selected cases are given.

REFERENCES.

[1] J. L. Kantor (New York), " A Clinical Study of Some Common Anatomical Abnormalities of the Colon," *Amer. Journal of Radiology*, 1924, ii. 414.

[2] Carman, *The Roentgen Diagnosis of Diseases of the Alimentary Tract*. London: Saunders, 1917.

[3] Meyer, *Roentgen Diagnostik in der Chirurgie*.

## Figures and Tables

**Case 1. f1:**
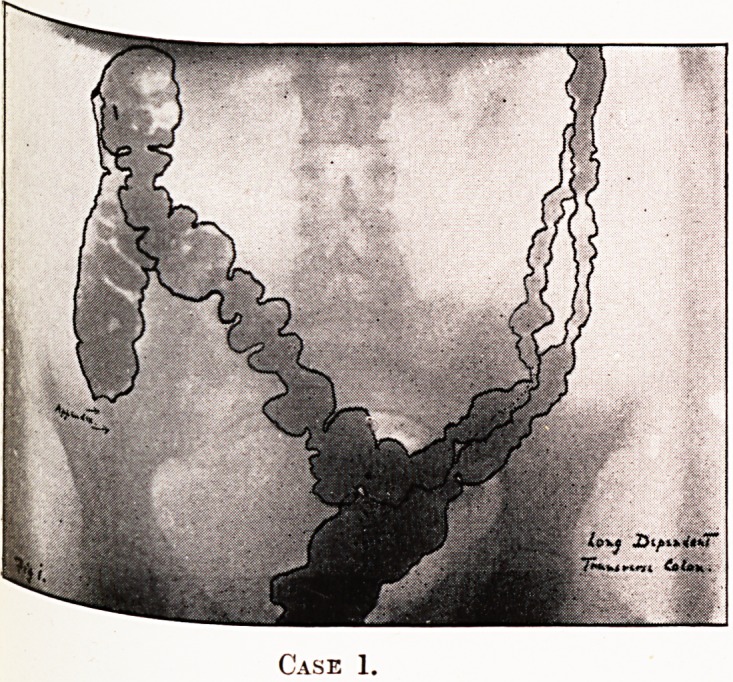


**Case 2. f2:**
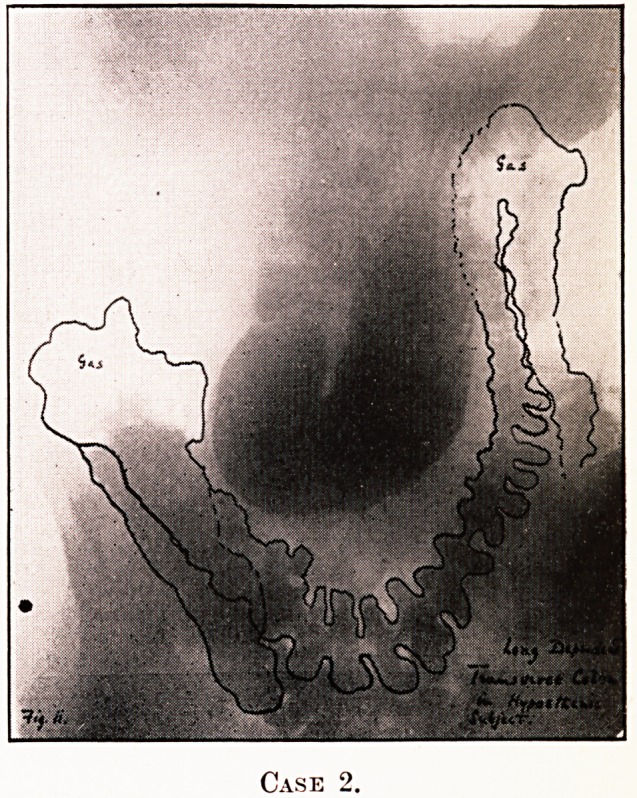


**Case 3. f3:**
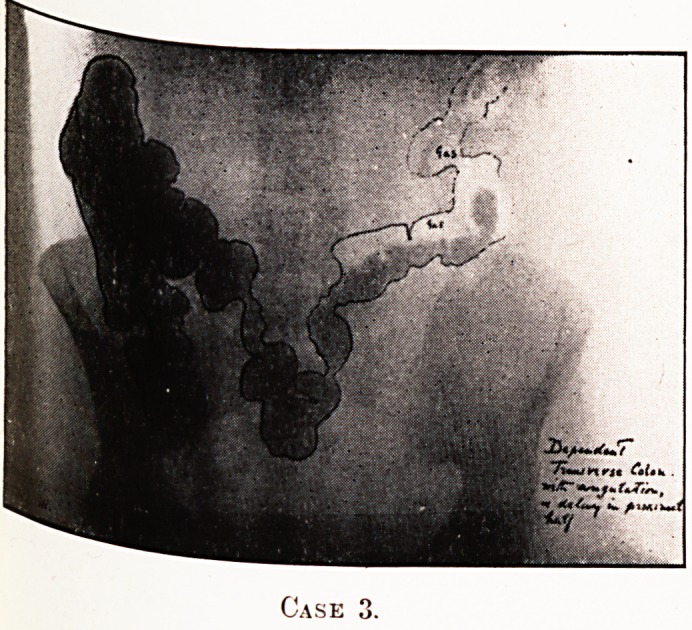


**Case 4. f4:**
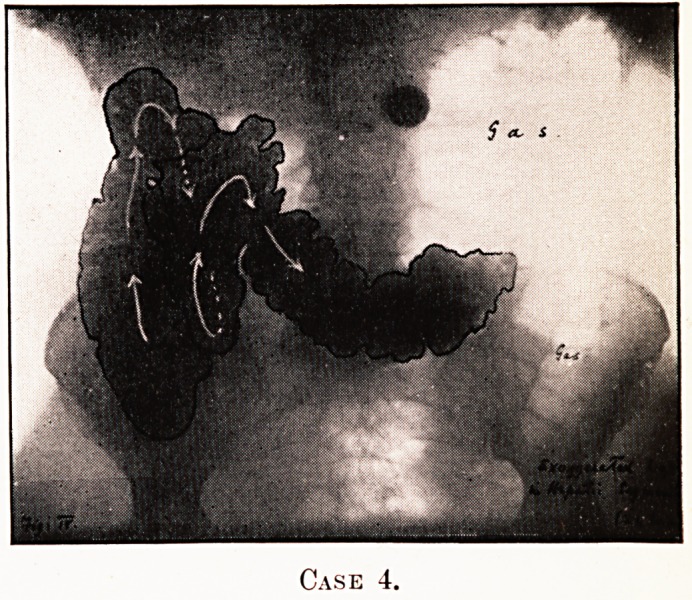


**Case 4. f5:**
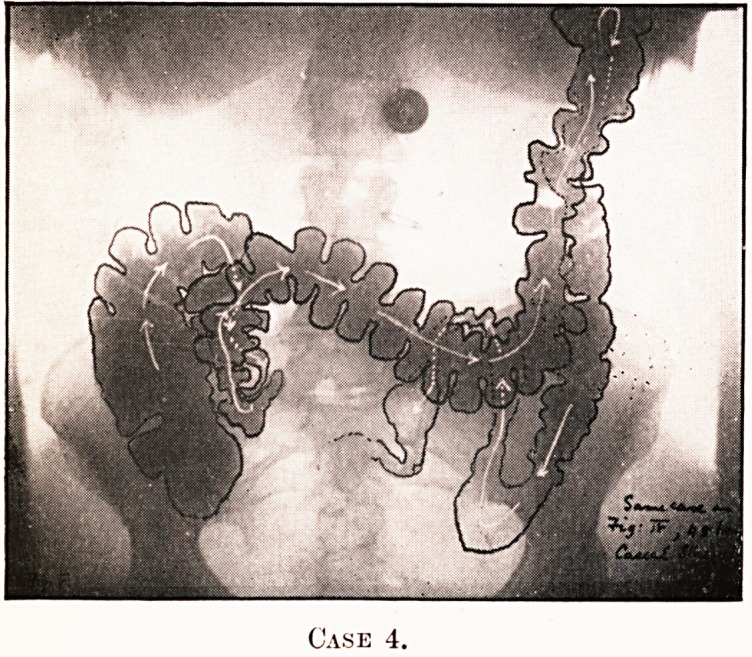


**Case 6. f6:**
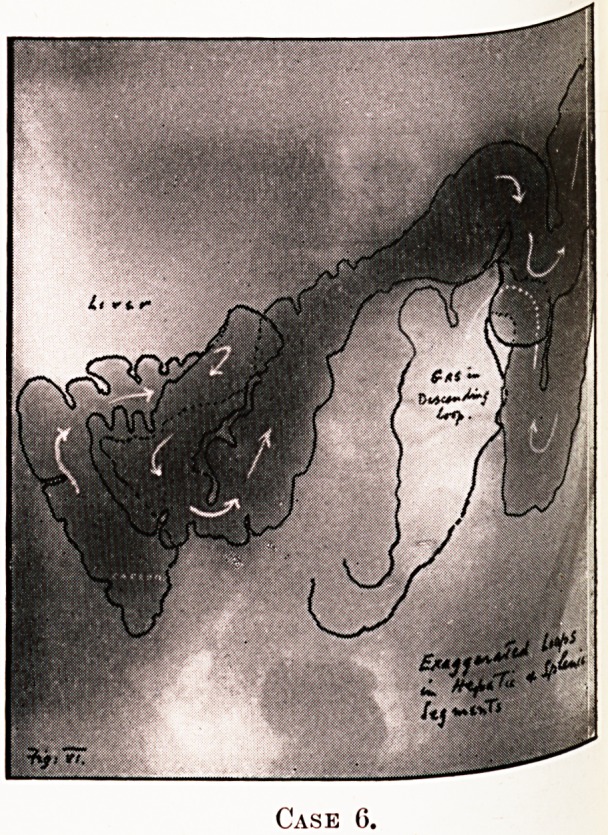


**Case 7. f7:**
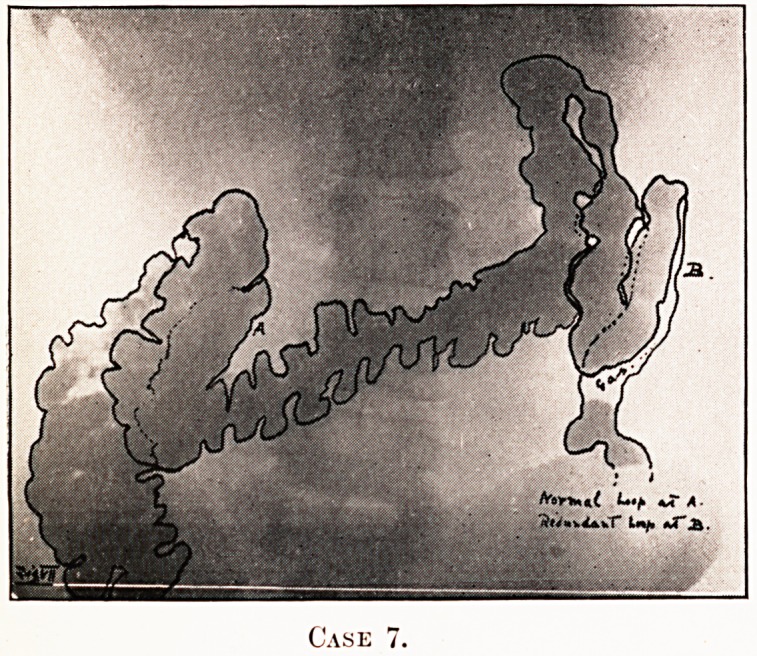


**Case 8. f8:**